# Effect of *Acacia concinna* Extract on Apoptosis Induction Associated with Endoplasmic Reticulum Stress and Modulated Intracellular Signaling Pathway in Human Colon HCT116 Cancer Cells

**DOI:** 10.3390/nu16213764

**Published:** 2024-11-01

**Authors:** Pornnapa Sitthisuk, Sukanda Innajak, Watcharaporn Poorahong, Siritron Samosorn, Kulvadee Dolsophon, Ramida Watanapokasin

**Affiliations:** 1 Department of Biochemistry, Faculty of Medicine, Srinakharinwirot University, Bangkok 10110, Thailand; pornnapa.sitthisuk@gmail.com (P.S.); suinnajak@gmail.com (S.I.); 2 Department of Biochemistry, Faculty of Medicine, Bangkok Thonburi University, Bangkok 10170, Thailand; noi.poorahong@gmail.com; 3 Department of Chemistry and Center of Excellence for Innovation in Chemistry, Faculty of Science, Srinakharinwirot University, Bangkok 10110, Thailand; siritron@g.swu.ac.th (S.S.); kulvadee@g.swu.ac.th (K.D.)

**Keywords:** *Acacia concinna*, apoptosis, colon cancer, KRAS mutant

## Abstract

Background: Colorectal cancer (CRC) stands as one of the most prevalent cancer types and among the most frequent causes of cancer-related death globally. *Acacia concinna* (AC) is a medicinal and edible plant that exhibits a multitude of biological properties, including anticancer properties. This study aimed to investigate the impact of the AC extract on apoptosis induction and the underlying mechanisms associated with this effect in KRAS-mutated human colon HCT116 cells. Methods: The effect of AC extract on cell cytotoxicity was evaluated using MTT assay. Nuclear morphological changes were visualized with Hoechst 33342 staining, while mitochondrial membrane potential (MMP) was assessed via JC-1 staining. Flow cytometry was employed for cell cycle analysis, and intracellular ROS levels were determined using DCFH-DA staining. Results: The results showed that HCT116 cells exposed to AC extract showed reduced cell growth and prompted apoptosis, as indicated by an increase in chromatin condensation, apoptotic bodies, the sub-G1 apoptotic cell population, and disrupted MMP. Expression levels of apoptosis mediator proteins determined by Western blot analysis showed an increase in pro-apoptotic proteins (Bak and Bax) while decreasing anti-apoptotic proteins (Bcl-2, Bcl-xL, and Mcl-1), leading to caspase-7 activation and PARP inactivation. AC extract was also found to enhance intracellular reactive oxygen species (ROS) levels and stimulate endoplasmic reticulum (ER) stress. Furthermore, AC extract increases the phosphorylation of ERK1/2, p38, and c-Jun while downregulating PI3K, Akt, β-catenin, and their downstream target proteins. Conclusions: These results demonstrate that AC extract could inhibit cancer cell growth via ROS-induced ER stress associated with apoptosis and regulate the MAPK, PI3K/Akt, and Wnt/β-catenin signaling pathways in HCT116 cells. Therefore, AC extract may be a novel candidate for natural anticancer resources for colon cancer treatment.

## 1. Introduction

Colorectal cancer (CRC) is a malignancy that develops in the colon or rectum, typically arising due to a multi-step process during which cells acquire a series of mutations [1]. Activating mutations in the oncogene Kirsten rat sarcoma (KRAS) are common, and present in up to 40% of CRC patients. These mutations are associated with enhanced cell survival and proliferation as well as reduced apoptosis, and often contribute to resistance against both conventional and targeted chemotherapies [2,3]. CRC is the second most common cause of cancer-related deaths worldwide and the third most diagnosed type of cancer [4]. According to GLOBOCAN 2020, approximately 1.15 million new cases of colon cancer and 0.58 million deaths were estimated worldwide. The incidences and mortality rates of CRC are constantly increasing due to lifestyle and other factors, with predictions of new cases rising to 1.92 million by 2040 [5]. CRC is primarily treated by surgical removal in the early stages; however, many cases are diagnosed at later stages, leading to chemotherapy treatment in advanced cases. The common chemotherapy drugs used to treat CRC, often in combination, include 5-fluorouracil (5-FU), oxaliplatin, and irinotecan. Nevertheless, the efficacy of chemotherapy drugs is limited due to their side effects on normal cells and the emergence of drug resistance in cancer cells [6]. Thus, the search for new anticancer agents is necessary for effective cancer treatment.

Currently, plant-derived phytochemicals offering health benefits and disease prevention, including cancer, have been extensively investigated for their potential as anticancer drugs [7]. *Acacia concinna* is a traditional medicinal plant belonging to the Fabaceae family and is widely found in Asian countries, including India, Sri Lanka, Myanmar, and Thailand. It has various common names, such as Soap-Pod and Shikakai, and is well-known as “Sompoi” in Thai [8,9]. *A. concinna* is used as a shampoo and in traditional medicine for its anti-dermatophyte and antimicrobial properties in treating skin disorders [10,11]. The plant contains various chemical components, predominantly saponins, flavonoids, and monoterpenoids [9,12], and exhibits numerous pharmacological properties, including antioxidant, anti-tyrosinase activity [10], and anticancer activity [13,14]. However, the mechanisms underlying the anticancer effects of *A. concinna* extract on colon cancer remain unknown.

Apoptosis is a type of programmed cell death occurring during normal processes such as embryonic development, aging, and the removal of damaged cells, thereby maintaining cellular homeostasis [15]. Morphological characteristics of apoptosis include cell shrinkage, chromatin condensation, membrane blebbing, nuclear fragmentation, and the creation of apoptotic bodies, which are phagocytosed by neighboring cells or phagocytes. This process does not trigger any inflammatory reactions and is not harmful to the host [16,17]. The morphological changes occur consecutively to apoptotic signaling events in response to stimuli such as DNA damage and ER stress. During apoptosis, the permeability of mitochondria was disrupted due to the action of the pro-apoptotic Bcl-2 family protein and the consequent release of cytochrome c, leading to caspase-3/7 activation, which in turn cleave several key enzymes, such as poly (ADP-ribose) polymerase (PARP), resulting in irreversible apoptosis cell death [18,19]. Thus, apoptosis serves as a major mechanism of cancer therapy and has become a famous target in various treatment strategies. Additionally, many anticancer drugs exert their anticancer effects by inducing apoptosis [20,21].

Various signaling pathways affect apoptosis. The mitogen-activated protein kinase (MAPK) pathways include three main sub-families: extracellular regulated kinase (ERK), p38 kinase (p38), and Jun N-terminal kinase (JNK), which regulate a variety of biological functions, including cell growth, differentiation, and death. According to previous research, ERK1/2, p38, and JNK are important modulators of both pro-apoptotic and anti-apoptotic protein activities [22]. Meanwhile, the phosphoinositide 3-kinase (PI3K)/protein kinase B (PKB, Akt) pathway is involved in cell survival by inhibiting apoptosis via suppressing the expression of pro-apoptotic signals such as FOXO and Bad [23]. In addition, activated Akt has the capability to stimulate the Wnt/β-catenin pathway [24]. Meanwhile, activation of the Wnt/β-catenin pathway results in the suppression of apoptosis by inhibiting caspase family activity, releasing cytochrome c, and decreasing the Bax/Bcl-2 expression ratio [25].

Therefore, in this study, we investigate the effects of *A. concinna* extract on cytotoxicity and apoptosis induction in the KRAS-mutated human colon HCT116 cell line and the underlying molecular mechanism by checking various cellular signaling pathways like ER stress, MAPK, PI3K/Akt, and Wnt/β-catenin. This study presented the potential anti-colon cancer activity of *A. concinna* that may be developed as an anticancer drug or combined with other drugs for colon cancer therapies in the future.

## 2. Materials and Methods

### 2.1. Materials

The materials for maintenance of cells; RPMI 1640 medium (Roswell Park Memorial Institute), penicillin-streptomycin, trypsin-EDTA, and FBS (fetal bovine serum) were acquired from HiMedia (HiMedia, Laboratories, Mumbai, India). MTT or 3-(4,5-dimethylthiazol-2-yl)-2,5-diphenyltetrazolium bromide was purchased from Sigma-Aldrich (St. Louis, MO, USA), while Hoechst 33342 dye [2′-(4-Ethoxyphenyl)-6-(4-methyl-1-piperazinyl)-1H,3′H-2,5′-bibenzimidazole] was obtained from Thermo Fisher Scientific (Invitrogen^TM^, Thermo Fisher Scientific Inc., Waltham, MA, USA). JC-1 dye (5,5′,6,6′-Tetrachloro-1,1′,3,3-tetraethylbenzimidazolylcarbocyanine iodide) was purchased from Sigma-Aldrich (Merck KGaA, Darmstadt, Germany). DCFH-DA dye (dichlorodihydrofluorescein diacetate), Guava Cell Cycle^®^ reagent, Immobilon™ Western Chemiluminescent HRP Substrate, and an anti-β-actin antibody were purchased from Merck Millipore (Merck Millipore Corp., Darmstadt, Germany). The antibodies used in Western blot analysis including anti-mouse/-rabbit primary antibody and HRP-conjugated secondary antibodies, were purchased from Cell Signaling (Cell Signaling Technology, Danvers, MA, USA) and Thermo Fisher Scientific, Inc. (Invitrogen^TM^, Waltham, MA, USA).

### 2.2. Plant Extraction

*A. concinna* pods were purchased from the Thai Lanna Herbal Industry, Chiang Mai province, Thailand, in September 2018. The pods were dried and ground into powder. Then, the powder (50 g) was extracted by maceration in 95% ethanol (500 mL) for 3 days in the dark at room temperature. After that, the extract was filtered through filter cloth and Whatman^®^ Qualitative Filter Paper No. 4, followed by evaporation to remove ethanol by using a rotary vacuum evaporator. The ethanol extract of *A. concinna* (AC) was dried using a vacuum desiccator to give the extract 11.36 g (22.72%). The AC extract was then stored at −20 °C in the dark until used in the experiment. The AC extract was dissolved in DMSO.

### 2.3. NMR and MS Analysis

The main components of the AC ethanol extract were characterized by liquid chromatography coupled to electrospray ionization mass spectrometry (LC-ESI-MS), proton nuclear magnetic resonance (^1^H NMR), carbon nuclear magnetic resonance (^13^C NMR), gradient correlation spectroscopy (gCOSY), gradient heteronuclear single quantum correlation (gHSQC), gradient heteronuclear multiple bond correlation (gHMBC), and diffusion ordered spectroscopy (DOSY). LC-ESI-MS analyses were run on a Thermo Scientific Dionex UltiMate 3000 Ultra-High Performance Liquid Chromatography system equipped with an electrospray ionization (ESI) source, an on-line degasser, a quaternary pump, a thermostatted column compartment, and an autosampler. Mass spectrometric detection was performed by a Bruker micrOTOF II. Separation was achieved on a C18 column 2.1 × 150 mm, 3 µm (Thermo Scientific, Waltham, MA, USA). The mobile phases consisting of solvent A (0.1% formic acid in water) and solvent B (acetonitrile) were used for the gradient elution. The ^1^H and ^13^C NMR spectra were recorded on a 500 MHz Bruker Avance NMR spectrometer, in DMSO-*d6* as solvent, and referenced to the solvent peak at 2.50 and 39.5 ppm, respectively.

### 2.4. Cell Culture and Maintenance

The human colon cancer cell line, HCT116 was obtained from the American Type Culture Collection (ATCC, Manassas, VA, USA). Cells were cultured in RPMI 1640 Medium containing 10% FBS, penicillin (100 U/mL), and streptomycin (100 µg/mL). The cells were maintained in a CO_2_ incubator at 37 °C under 5% carbon dioxide (CO_2_) and saturated humidity (95%). Sub-culturing of the cells was performed every 2–3 days.

### 2.5. MTT Assay

The cytotoxicity of AC extract was evaluated using the MTT assay. Cells were seeded in 96-well culture plates and incubated overnight. Following incubation, the cells were then treated with AC extract at 0, 25, 50, 100, 200, 300, 400, and 500 µg/mL, while the control cells received 0.25% DMSO for 24 h. Subsequently, the supernatant was removed, and MTT solution (0.5 mg/mL) was added. The culture plates were then incubated for 2 h at 37 °C. Following incubation, DMSO was applied to solubilize the formazan crystals. The absorbance was then measured at 570 nm using a microplate reader (Multiskan Sky Microplate Spectrophotometer, Waltham, MA, USA). Cell viability percentages (%) were calculated in comparison to the control group, and the IC_50_ values were performed in GraphPad Prism 9 Software (GraphPad Prism Software, Inc., San Diego, CA, USA).

### 2.6. Nuclear Morphological Changes Detection

Hoechst 33342 staining was performed to assess chromatin condensation. Briefly, cells were seeded and treated with AC extract at 0, 50, 100, 200, and 250 µg/mL, whereas the control cells received 0.25% DMSO for 24 h. The cells were then incubated for 30 min after being stained with Hoechst 33342 fluorescent dye. The images were visualized by using a fluorescence microscope (DP73+IX71 Olympus, Tokyo, Japan).

### 2.7. Measurement of Mitochondrial Membrane Potential (MMP)

JC-1 staining was utilized to evaluate the effect of AC extract on MMP. Cells were seeded and treated with AC extract at 0, 50, 100, 200, and 250 µg/mL, and the control group received 0.25% DMSO for 9 h, then the cells were incubated with JC-1 fluorescence dye at room temperature for 10 min. The images were examined under a fluorescence microscope (DP73+IX71 Olympus, Tokyo, Japan).

### 2.8. Cell Cycle Analysis

Flow cytometry was utilized to assess the impact of AC extract on cell cycle distribution. In brief, HCT116 cells were seeded and treated with AC extract at 0, 50, 100, 200, and 250 µg/mL. The control group received 0.25% DMSO for 12 h. After collecting the cells, 70% cold ethanol was used to fix the cells, and they were subsequently stained with Guava Cell Cycle^®^ reagent (Merck Millipore Corporation, Merck KGaA, Darmstadt, Germany). The stained cells were then assessed for DNA content using the Guava EasyCyte^TM^ flow cytometer and GuavaSoft^TM^ software version 3.2 (Merck Millipore Corporation, Merck KGaA, Darmstadt, Germany).

### 2.9. Intracellular ROS Measurement

HCT116 cells were seeded and treated with AC extract at 0, 50, 100, 200, and 250 µg/mL, and the control group received 0.25% DMSO. They were then incubated with 20 μM DCFH-DA fluorescence dye at 37 °C for 30 min. After incubation, stained cells were examined using a fluorescence microscope (DP73+IX71, Olympus, Tokyo, Japan).

### 2.10. Western Blot Analysis

Protein expression was evaluated by Western blot analysis. The cells were collected, and RIPA lysis buffer (250 mM NaCl, 50 mM Tris-HCl, pH 7.5, 5 mM EDTA, 0.5% Triton X-100, protease inhibitor cocktail, and 10 mM PMSF) was utilized for extracting the total protein from the cells. Following SDS-PAGE separation, the protein mixture was transferred to PVDF (polyvinylidene fluoride) membranes. The membranes were subsequently blocked with blocking buffer for 1 h, incubated with primary antibodies targeting the specific proteins of interest at 4 °C overnight, and incubated with HRP-conjugated secondary antibodies for 1 h, respectively. The immunoreactivity protein bands were exposed by using chemiluminescent HRP substrate (ECL) and visualized using a gel documentary machine (AllianceQ9 advanced, Cambridge, UK). ImageJ software version 1.53e was used to quantify the intensity of the protein bands, and the results were presented as the ratio relative to the intensity of β-actin.

### 2.11. Statistical Analysis

The data were expressed as the mean ± standard deviation (SD). Statistically significant differences between groups were evaluated using one-way analysis variance (ANOVA), and Tukey’s post hoc test was then employed. Statistical analysis was performed using SPSS statistical software package version 20.0 (IBM Crop., Albany, NY, USA). Statistical significance was defined as *p* < 0.05 and 0.01.

## 3. Results

### 3.1. NMR and MS Analysis of AC Extract

**Figure 1:**
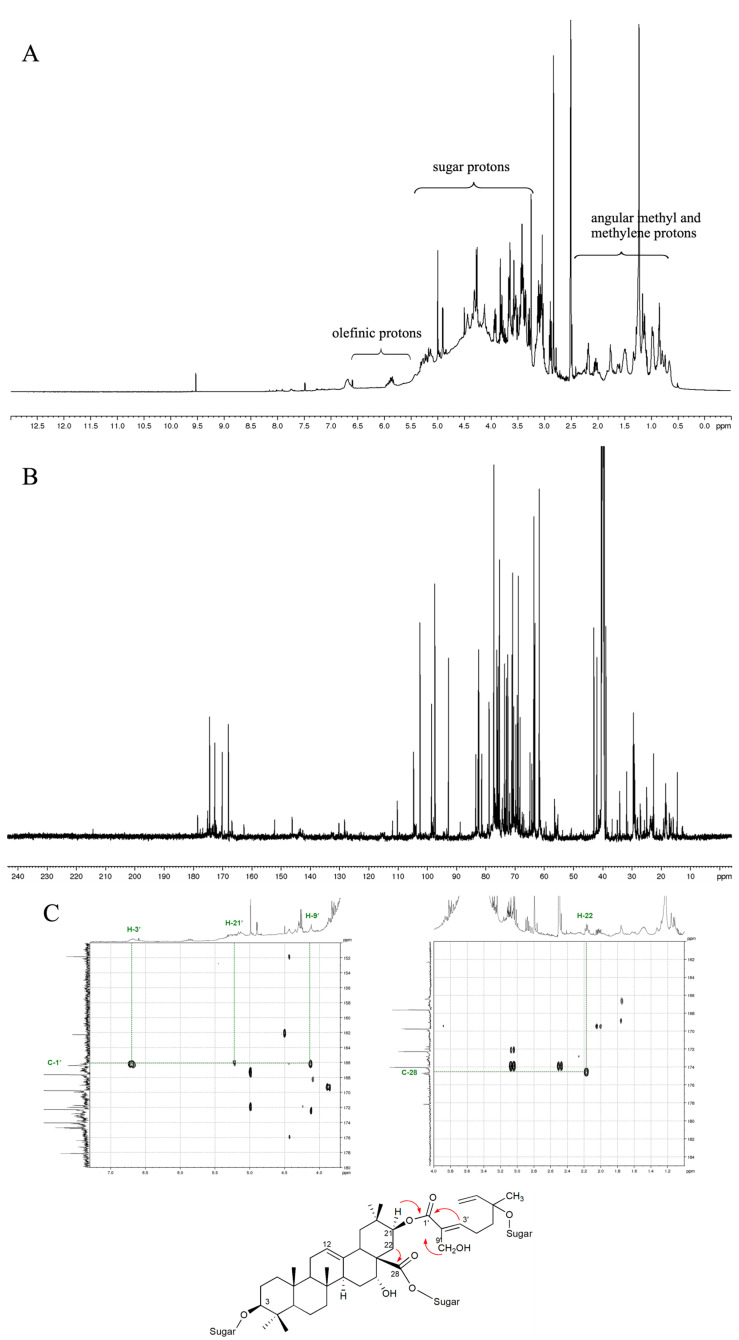
NMR spectra of the AC ethanol extract in DMSO-*d6*: (**A**) ^1^H NMR spectrum; (**B**) ^13^C NMR spectrum; (**C**) HMBC correlations.

The LC-ESI-MS spectrum of the AC ethanol extract indicated the presence of flavonoids in comparison with the previous report on Acacia pod extracts [26]. The main peaks showed [M + Na]^+^ ion with *m*/*z* 325.2 and [M + H]^+^ ion with *m*/*z* 319.2 corresponding to pentahydroxyflavone and hexahydroxyflavone, respectively. In order to obtain more data on chemical compositions in the AC ethanol extract, 1D and 2D NMR spectroscopic analyses were performed. Even though the signals in the ^1^H NMR spectrum (Figure 1A) overlapped, some information was discernible. The presence of angular methyl and methylene protons, sugar protons, and olefinic protons signals in the range of δ 0.7–2.4, 3.0–5.0, and 5.5–6.5, respectively, could be observed. The ^13^C NMR (Figure 1B) indicated anomeric carbons, olefinic carbons, and carbonyl carbons signals in the region of 93–112, 121–145, and 162–215, respectively. Comparing the NMR data with the previous report [13] suggested that acacic acid-type saponins were in the extract. The HMBC experimental data indicated that the methylene proton signal observed at δ 2.17 (H-22) correlated with the carbonyl carbon signal at δ 174.5 (C-28). Moreover, the methine proton signal at δ 5.25 (H-21), olefinic proton signal at δ 6.70 (H-3′), and methylene proton signal at δ 4.12 (H-9′) correlated with the carbonyl carbon signal at δ 166.1 (C-1′). This clearly indicates that the extract contained acacic acid-type saponins (Figure 1C).

### 3.2. AC Extract Reduces Cell Viability in HCT116 Cells

**Figure 2:**
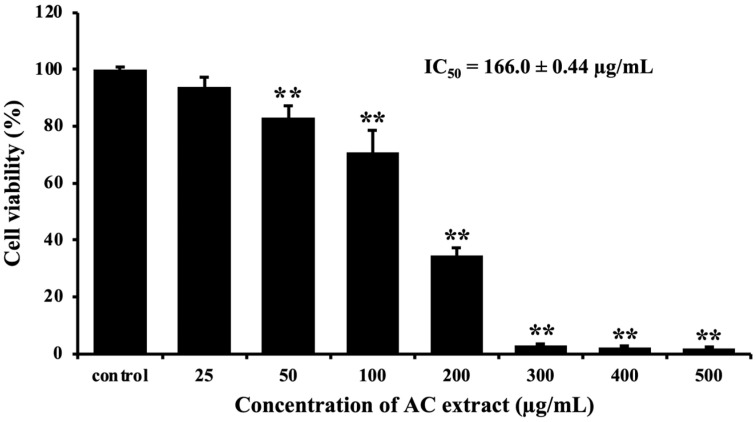
AC extract inhibited cell proliferation in HCT116 cells. The cells were treated with different concentrations of AC extract for 24 h and examined using the MTT assay. The cell viability is presented as a percentage compared to the control cells. ** *p* < 0.01, indicating significant differences compared to the control.

First, we determined whether AC extract can inhibit the proliferation of HCT116 cells by using the MTT assay. We found that AC extract significantly reduced cell viability on HCT116 cells with an IC_50_ value of 166.0 ± 0.44 µg/mL (Figure 2). In addition, we tested the effect of AC extract in normal cells (HaCat cells), and the IC_50_ value was 371.7 ± 1.477 µg/mL (Figure S3). This result suggested that AC extract could inhibit the proliferation of HCT116 cells.

### 3.3. AC Extract Induces Apoptosis in HCT116 Cells

**Figure 3:**
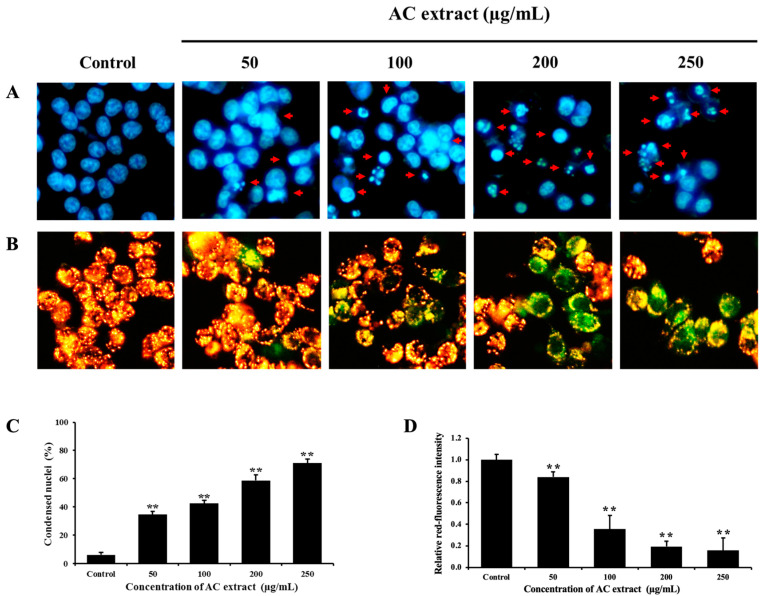
Effect of AC extract on the induction of apoptosis in HCT116 cells. (**A**) Nuclear morphological changes were assessed by staining cells with Hoechst 33342 and observed by fluorescence microscopy (20×). The red arrows indicated nuclear condensation and apoptotic bodies. (**B**) The loss of MMP in the cells was evaluated using JC-1 staining and observed by fluorescence microscopy (20×). The green fluorescence indicated the loss of MMP. (**C**) The histogram represented the percent of nuclear-condensed cells relative to the control cells. (**D**) The histogram represented the relative intensity of red fluorescence compared to the control cells. ** *p* < 0.01, indicating significant differences compared to the control.

Most anticancer drugs have properties that induce apoptosis [20]; thus, we next determine whether the proliferation inhibitory effect is associated with apoptosis induction. The nuclear morphological changes and MMP detection, which are major characteristics of apoptosis, were carried out using Hoechst 33342 and JC-1 staining, respectively. As shown in Figure 3A, after treatment for 24 h, the AC-treated cells have smaller, brighter nuclei than the control group, indicating nuclear and cytoplasmic condensation in a dose-dependent manner. Cell shrinkage as well as apoptotic bodies were also observed. Additionally, AC extract could induce the loss of MMP in the HCT116 cells demonstrated by the reduced red fluorescence of JC-1 dye (Figure 3B). This result revealed that AC treatment led to mitochondrial dysfunction. Taken together, our findings demonstrate that AC extract inhibited the proliferation of HCT116 cells through apoptosis induction.

### 3.4. AC Extract Increased Population of HCT116 Cells in Sub-G1 Phase

**Figure 4:**
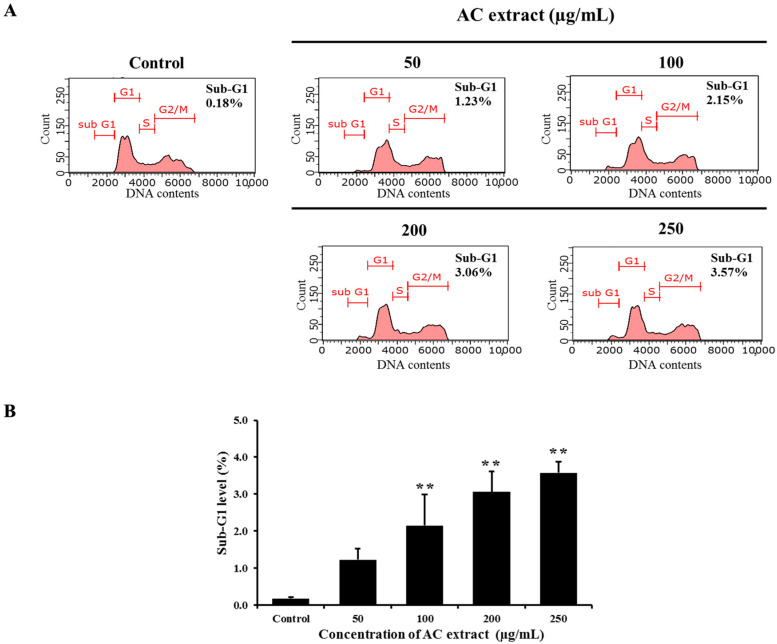
Effect of AC extract on cell cycle distribution in HCT116 cells. (**A**) The histograms represent the DNA content analysis performed by flow cytometry. (**B**) The graphical representation compared the relative sub-G1 level to the control cells. ** *p* < 0.01, indicating significant differences compared to the control.

The apoptosis-inducing effect of AC extract in HCT116 cells was confirmed by cell cycle analysis. The histograms in Figure 4 show the cell cycle distribution of HCT116 cells, implying that treatment of the cells with AC extract led to an increase sub-G1 population, indicating the presence of apoptotic cells. The percentages of cells significantly increased from 0.18% observed in the control cells to 1.23, 2.15, 3.06, and 3.57% at concentrations of 50, 100, 200, and 250 µg/mL of AC extract, respectively. These findings clearly demonstrated that AC extract induced apoptosis in HCT116 cells.

### 3.5. AC Extract Induced Intracellular ROS Production

**Figure 5:**
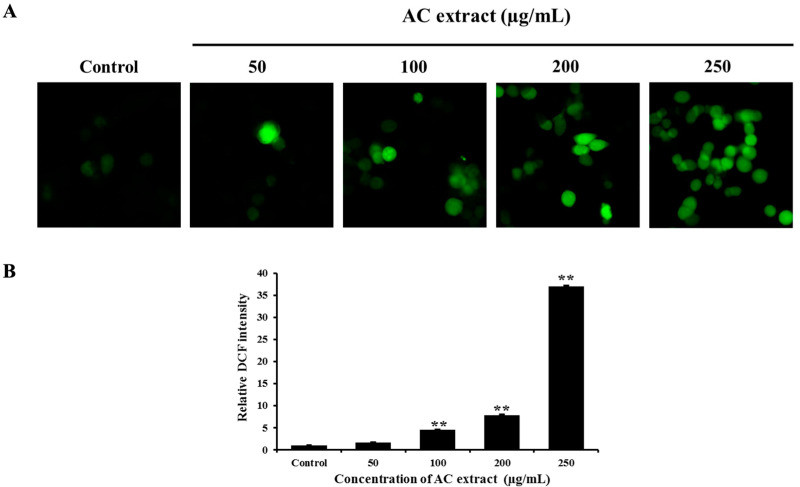
Effect of AC extract on intracellular ROS level in HCT116 cells. The cells were treated with AC extract for 2 h, determined by DCFH-DA staining, and observed by a fluorescence microscope (20×). (**A**) DCF fluorescence image in HCT116 cells. The green fluorescence indicated ROS formation in the cells. (**B**) The relative DCF fluorescence intensity compared to the control cells. ** *p* < 0.01, indicating significant differences compared to the control.

The accumulation of ROS has been associated with the disruption of MMP [27] considered a trigger for the mitochondrial apoptotic pathway. While our results showed that AC extract caused a loss of MMP, for this reason, the level of intracellular ROS was measured using DCFH-DA staining. As shown in Figure 5, AC extract significantly increased the level of intracellular ROS in HCT116 cells after 2 h of incubation with AC extract as compared to the control cells.

### 3.6. Effect of AC Extract on Apoptotic-Related Protein Expression

**Figure 6:**
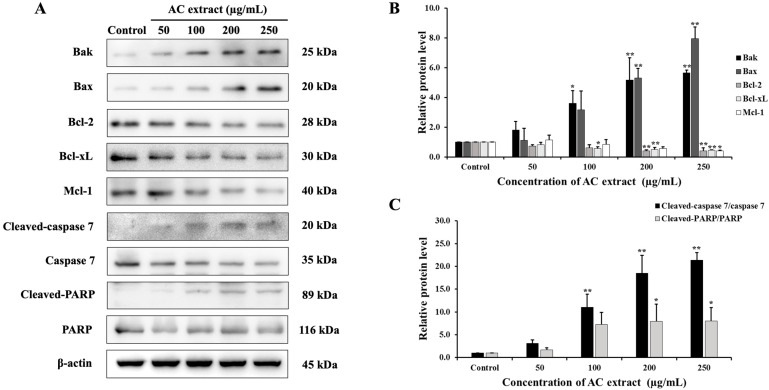
Effect of AC extract on apoptosis-related protein expression in HCT116 cells. (**A**) The expression of protein was detected by Western blot analysis. (**B**,**C**) The relative band intensity of apoptosis-related proteins compared to the control group. * *p* < 0.05 and ** *p* < 0.01, indicating significant differences compared to the control.

To explicate the molecular mechanisms of AC extract-induced apoptosis, the expression of several key apoptotic-related proteins was examined. The treatment with AC extract for 24 h induced a significant decrease in the anti-apoptotic proteins B-cell leukemia/lymphoma 2 (Bcl-2), B-cell lymphoma-extra-large (Bcl-xL), and myeloid leukemia 1 (Mcl-1), while a significant increase in the pro-apoptotic proteins Bcl2-associated X protein (Bax) and Bcl-2-antagonist/killer (Bak) resulted in the activation of the cleaved form of cysteine-dependent aspartate-specific protease (caspase-7). Furthermore, the level of a DNA repair enzyme, cleaved-PARP (inactive form), was also increased in AC-treated HCT116 cells (Figure 6). Therefore, these results indicated that the mechanism of apoptosis induction in HCT116 cells by AC extract was involved in the activation of caspase-dependent apoptosis via the mitochondrial pathways.

### 3.7. Effect of AC Extract on ER Stress

**Figure 7:**
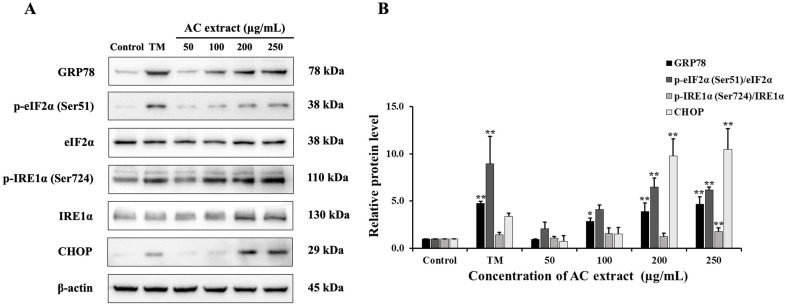
Effect of AC extract on ER stress-related proteins in HCT116 cells. The cells were treated with AC extract or Tm (10 µg/mL). (**A**) The protein expression was examined by Western blot analysis. (**B**) The relative band intensity compared to the control group. * *p* < 0.05 and ** *p* < 0.01, indicating significant differences compared to the control.

A previous study indicated that an increase in ROS levels can cause ER stress and lead to cell death [28]. We therefore conducted Western blot analysis to further elucidate the possible apoptotic pathway induced by AC extract associated with the ER stress. As shown in Figure 7, AC extract upregulated the expression of glucose-regulated protein 78 (GRP78), protein kinase RNA-like endoplasmic reticulum kinase (PERK)/eukaryotic initiation factor 2 alpha (eIF2α) pathway, leading to the increased C/EBP homologous protein (CHOP) in HCT116 cells. Moreover, AC extract increased the level of p-IRE1α (inositol-requiring enzyme 1 α). The protein expression patterns observed in AC-treated cells were similar to those in tunicamycin (Tm)-treated cells, a widely recognized inducer of ER stress. These results suggested that AC extract induced ER stress, which may be closely related to apoptosis induction in HCT116 cells.

### 3.8. Effect of AC Extract on MAPK Signaling Pathway

**Figure 8:**
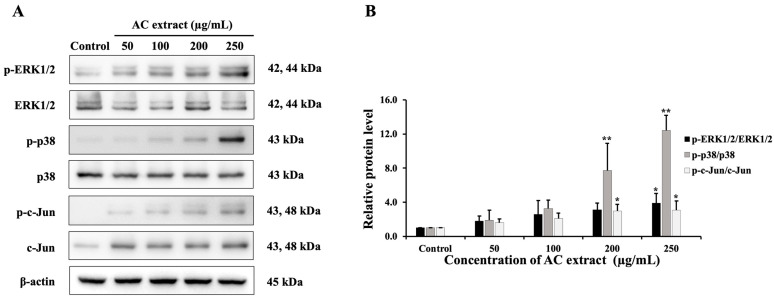
Effect of AC extract on MAPK pathway in HCT116 cells. (**A**) The protein expression was detected by Western blot analysis. (**B**) The relative band intensity compared to the control group. * *p* < 0.05 and ** *p* < 0.01, indicating significant differences compared to the control.

The MAPK pathway downstream of KRAS plays a major role in cell differentiation, proliferation, and apoptosis [22]. Therefore, we studied the protein levels of this pathway. The results, shown in Figure 8, indicated that AC extract increased the ratio of the phosphorylated form to the total form of ERK1/2, p38, and c-Jun proteins. These results revealed that AC extract may inhibit HCT116 cell growth by regulating the MAPK pathway.

### 3.9. Effect of AC Extract on PI3K/Akt and Wnt/β-Catenin Signaling Pathways

**Figure 9:**
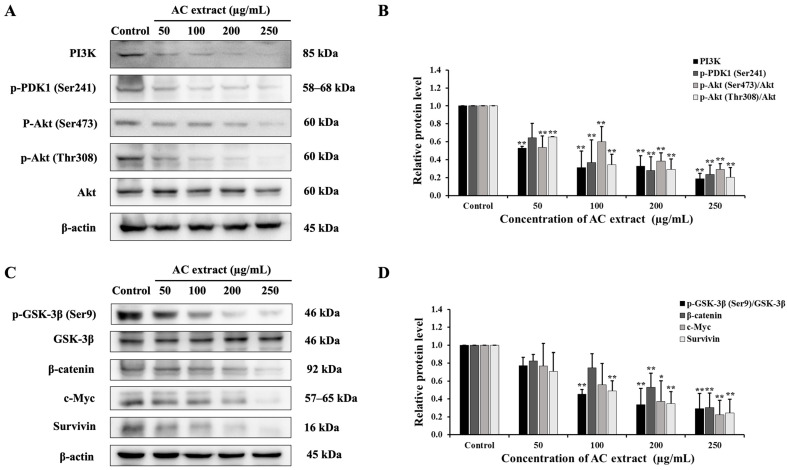
Effect of AC extract on PI3K/Akt and Wnt/β-catenin signaling pathway in HCT116 cells. (**A**) The expression of PI3K, p-PDK1, p-Akt (Ser473), p-Akt (Thr308), and Akt. (**B**) The relative band intensity of PI3K/Akt proteins compared to the control group. (**C**) The protein expression of p-GSK-3β, GSK-3β, β-catenin, c-Myc, and survivin. (**D**) The relative band intensity of Wnt/β-catenin proteins compared to the control group. * *p* < 0.05 and ** *p* < 0.01, indicating significant differences compared to the control.

It is well-known that PI3K/Akt serves as a key signaling transduction pathway for regulating cellular survival and inhibiting apoptosis, downstream of KRAS [23]. Thereby, the effect of AC extract on this pathway in HCT116 cells was detected. The results found that AC extract decreased the levels of PI3K, p-PDK1 (3-phosphoinositide-dependent kinase 1), p-Akt (Ser473), and p-Akt (Thr308) (Figure 9A,B). The PI3K/Akt pathway contributes to the triggering of the Wnt/β-catenin pathway [24]; therefore, we next investigated the expression of this pathway. As shown in Figure 9C, AC extract downregulated the expressions of phospho-glycogen synthase kinase-3 beta (p-GSK-3β), the inactive form of GSK-3β, thereby enhancing β-catenin degradation, as evidenced by decreased β-catenin and their downstream target (c-Myc, survivin) expression. Thus, our results suggested that AC extract could induce apoptosis by suppressing the Wnt/β-catenin pathway by restraining the upstream kinase activity of the PI3/Akt pathway.

## 4. Discussion

Considerable research suggests the potential impact of bioactive compounds in the prevention and treatment of cancer [29]. Moreover, bioactive compounds from plants or herbs are generally safe and low toxic; this is an alternative approach to avoiding the side effects of synthetic medicines [30]. In this study, we determined whether AC extract has anticancer properties in human colon cancer cells. This study illustrated that AC extract exhibited a significant anti-proliferative effect in HCT116 cells. In a previous study using kinmoonosides A-C, novel saponins isolated from a methanolic extract of *A. concinna* pods also exhibited a significant cytotoxic effect against human HT-1080 fibrosarcoma cells [13], supporting our results and indicating that AC extract has anticancer potential in cancer cells.

Phytochemicals are bioactive compounds derived from plants that offer health benefits, including the prevention of diabetes, obesity, and cancer. These phytochemical compounds contain several groups, such as polyphenols, carotenoids, and saponins [31]. Flavonoids are a large family of polyphenols that exhibit anticancer effects by modulating multiple mechanisms of action such as apoptosis induction, cell cycle arrest, and invasiveness [32]. In this study, the phytochemical compounds of the AC extract detected as the flavonoid (Figure S1) were analyzed by NMR and MS. The main peaks of the MS profile of AC extract corresponded to pentahydroxyflavone, hexahydroxyflavone, and acacic acid-type saponins in comparison with the previous reports of Acacia pods extracts [13,26,33]. A previous study found that the methanolic extract from the *A. concinna* plant was found to contain flavonoids and phenols and showed cytotoxicity on human breast cancer cells, MCF-7 [11]. In addition, the extract containing triterpenoid saponins showed cytotoxicity against HT-1080 fibrosarcoma cells [13]. Therefore, the anticancer effect of the AC extract may be due to the flavonoids and saponins presented. However, further studies are necessary to isolate, purify, and test the active compounds of AC extract to assess their anticancer activity.

The main goals of cancer treatment are the inhibition of cancer cell growth and the elimination of cancer cells while providing low toxicity to normal cells. It is commonly known that most many chemotherapeutic agents can kill cancer cells by activating apoptotic pathways [21]. Therefore, we hypothesized that the inhibitory effect of AC extract on HCT116 cells may involve apoptosis induction. Our study revealed that AC extract selectively induced apoptosis in HCT116 cells indicated by chromatin condensation and loss of MMP as well as increased sub-G1 populations. The loss of MMP is recognized as a key step in the mitochondrial or intrinsic apoptosis pathway. The loss of MMP occurs by activation of pro-apoptotic protein. This event results in the activation of numerous caspases and cleavage of downstream death effector proteins [34]. The Western blot analysis of AC-treated cells confirmed such a hypothesis. We discovered that AC extract increased the protein expression of cleaved caspase-7 responsible for cleaving downstream substrates, PARP. The activation of effector caspases is well known to be responsible for the hallmarks of apoptosis cells, such as DNA fragmentation [35]. AC-treated cells also increased cleaved-PARP levels, which prevented the cancer cells from repairing damage required for their survival, and finally caused irreversible apoptosis cell death and complete elimination [18,19]. We also confirmed that AC extract regulated Bcl-2 family members, which initiated the apoptosis process in HCT116 cells via the intrinsic pathway. According to previous studies, morin (3,5,7,2′,4′-pentahydroxyflavone) stimulates caspases-8, -9, and -3, causes PARP cleavage, and modulates the Fas receptor and Bcl-2 family members, suggesting that it induces both the extrinsic and intrinsic apoptosis pathways in HCT 116 cells [36].

Several studies have suggested that elevated intracellular levels of ROS can trigger ER stress, potentially leading to mitochondrial dysfunction and ultimately initiating apoptosis [28,37]. Interestingly, AC extract has been found to induce intracellular ROS generation and ER stress by upregulating the expression of GRP78, p-eIF2α, CHOP, and p-IRE1α. Increased levels of GRP78/BiP have been reported to activate PERK by autophosphorylation and homomultimerization. Activated PERK subsequently phosphorylates eIF2α, leading to the activation of ATF4 and its downstream targets [38,39]. Normally, mild ER stress conditions can restore ER homeostasis through the unfolded protein response (UPR). Nevertheless, prolonged ER stress can trigger apoptosis via increased CHOP expression, which is mainly regulated through the PERK/eIF-2α/ATF-6 pathway [40]. The activation of CHOP is regarded as a key event for ER stress-induced apoptosis. Studies have shown that CHOP triggered apoptosis by suppressing anti-apoptosis proteins such as Bcl-2 and Mcl-1 and by enhancing the expression of the pro-apoptosis protein Bim. Subsequently, Bim regulated Bax and Bak, leading to disruption of the permeabilization of the mitochondrial outer membrane [41]. Previous investigation has shown that quercetin, a bioactive flavonoid induced apoptosis in human cervical cancer cells (HeLa) by ER stress induction. The levels of caspase-3, GRP78, p-PERK, c-ATF6, IRE1, and CHOP showed a progressive increase corresponding to the rising concentration of quercetin [42]. Another study showed that quercetin induced cell apoptosis, intracellular ROS production, and ER stress in prostate cancer cells (PC-3) [43]. In addition, a synthetic derivative of quercetin called TEF (5,3′-dihydroxy-3,7,4′-triethoxyflavone) also induced apoptosis and ER stress via the IRE1-α and mito-JNK pathways in HCT-116 cells [44]. Our results were consistent with their observations and demonstrated that AC extract induced ER stress via the GRP78/eIF-2α/CHOP pathway. Additionally, activated IRE1 can activate MAPK pathways including p38 and JNK, which regulate Bcl-2 family proteins [39,45]. Taken together, these results demonstrate that AC extract induced ER stress-mediated apoptosis in HCT116 cells. 

KRAS-mutant CRC cancer is linked to decreased survival and increased tumor aggressiveness [46]. Designing drugs that directly target mutant KRAS constitutes a significant challenge. Thus, an alternative strategy is to target downstream pathways such as the MAPK and Akt pathways [2]. Both p38 and JNK respond to both extracellular and intracellular stresses. Activation of p38 promotes cell apoptosis via the regulation of a variety of Bcl-2 family proteins, such as Bax, and the activating caspase family [47]. We reported that the expression of p-p38 and p-c-Jun were increased upon treatment with AC extract. c-Jun is one of the transcription factor AP-1 family members that can be phosphorylated by JNK. The activation of JNK may stimulate apoptosis by upregulating the transcription of pro-apoptotic genes such as the Fas/FasL pathway through c-Jun/AP-1 [48]. Previous studies have indicated that quercetin triggers apoptosis by activating the JNK pathway in KRAS-mutant colorectal cancer cells [2]. It has also been reported that the activation of ERK1/2 is linked to the induction of apoptosis. Kim S et al. 2019 reported the anti-tumor and apoptotic effects are elicited by quercetin through the regulation of MAPK pathways by increasing the levels of Bax, cleaved-PARP, p-JNK, p-p38, and p-ERK1/2, while reducing Bcl-2 in A375SM melanoma cells [49]. In the present study, AC extract increased the expression of p-ERK1/2 in HCT116 cells. Hence, AC-induced apoptosis in HCT116 cells may be related to MAPK pathway activation.

The PI3K/Akt pathway is widely recognized for regulating cell survival, cell proliferation, and cancer drug resistance. Mutations and activation of protein members within the PI3K/Akt/mTOR pathway are commonly found in cancer [50]. Several studies have indicated that plant extracts can inhibit this pathway, resulting in decreased cell proliferation and increased apoptosis. For instance, quercetin has shown anticancer effects via induced cell death in JAR and JEG3 choriocarcinoma cell lines through PI3K pathways by inhibiting p-Akt, p-P70S6K, and p-S6 proteins [51]. These findings correlated with our research, wherein we observed that AC extract reduced the expression of PI3K/Akt protein in HCT116 cells.

Increased Wnt/β-catenin signaling pathway activity is linked to carcinogenesis in CRC [52]. β-catenin is a key component of this pathway, which is regulated by a destruction complex. GSK3-β is part of the destruction complex that phosphorylates β-catenin in the cytosol leading to its degradation via the ubiquitin-proteasome system. GSK3β activation is regulated by Akt which phosphorylates GSK3β, thereby inactivating it and preventing the formation of the destruction complex [24,52,53,54]. As reported, we demonstrated that AC extract can inhibit both the PI3K/Akt and the Wnt/β-catenin pathway, which are crucial for tumor initiation and development. [55]. Similarly, quercetin has the potential to enhance apoptosis in human GBM T98G cells by inhibiting the Wnt3a/β-catenin pathway and the Akt/NF-κB signaling pathway [56].

## 5. Conclusions

In conclusion, this study suggested that AC extract can be considered a natural source of anticancer agents for colon cancer treatment. Our findings indicated that AC extract suppressed cell proliferation and apoptosis induction in HCT116 cells through various signaling pathways, including activation of the intrinsic caspase pathway, ROS-induced ER stress, and the MAPK pathway, while inhibiting the PI3K/Akt and Wnt/β-catenin pathways. This is the first report on the mechanism of apoptosis induction of AC extract in HCT116 cells. Therefore, further investigation may be required to explore the relationship between pathways to comprehensively understand the inhibitory effects of AC extract on HCT116 cells.

## Data Availability

The datasets analyzed during the current study are available from the corresponding author on reasonable request.

## Supplementary Materials

The following supporting information can be downloaded at: https://www.mdpi.com/article/10.3390/nu16213764/s1, Figure S1: Total ion chromatogram by positive mode electrospray ionization mass (A). ^1^H-NMR spectrum with expansion of AC extract in DMSO-*d6* (B).; Figure S2: The dried pods of *Acacia concinna* (A) were ground into powder (B) for extraction. The crude extract of *A. concinna* (C) was kept in the refrigerator, in the dark, until used in the experiment.; Figure S3: Effect of AC extract on cell viability in HaCat cells by MTT assay. The cell line was treated with various concentrations of AC extract for 24 h. The results were the mean values ± SD.
