# Supplementary material for: Effect of Acacia concinna Extract on Apoptosis Induction Associated with Endoplasmic Reticulum Stress and Modulated Intracellular Signaling Pathway in Human Colon HCT116 Cancer Cells
Source: Nutrients. 2024 Nov 1;16(21):3764. doi: 10.3390/nu16213764 (PMC11547357; doi:10.3390/nu16213764)
Supplement: Supplementary file 1 [file nutrients-16-03764-s001.zip › nutrients-3246784-supplementary.pdf]

Supplementary Figures

A

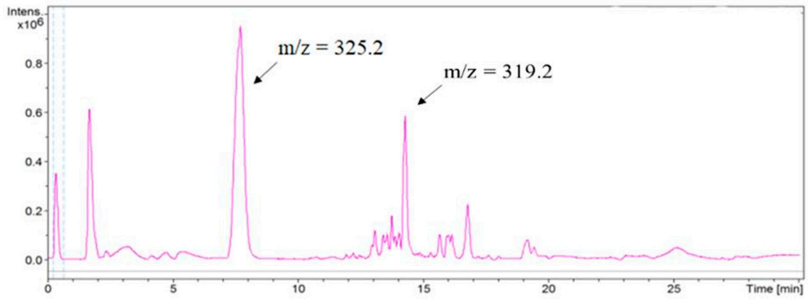

B

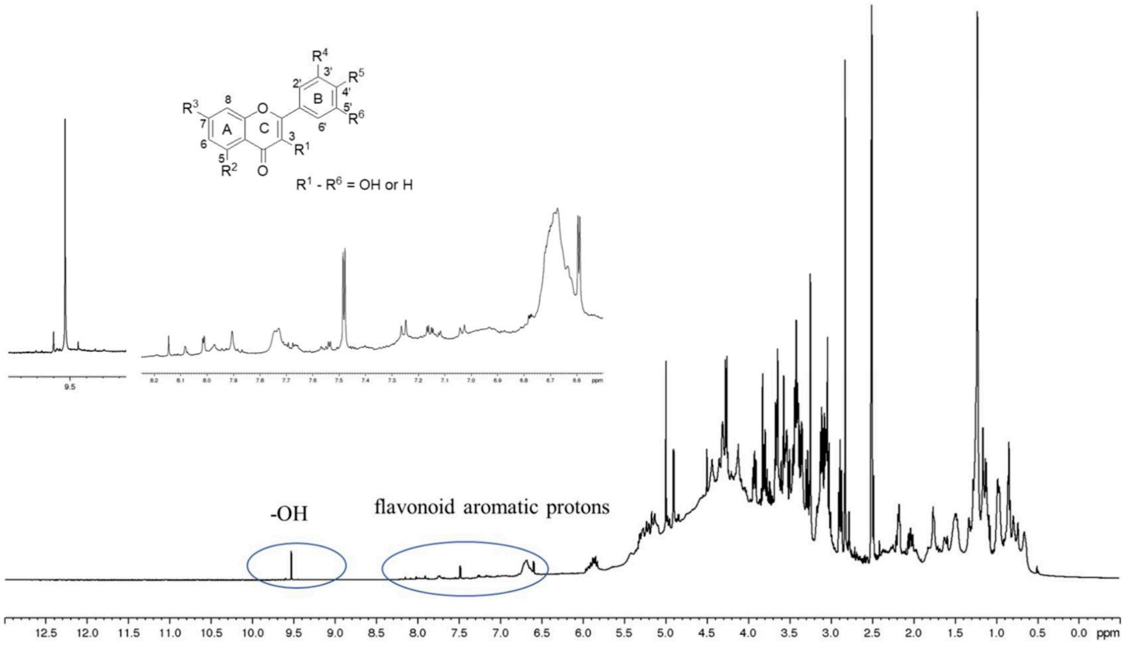

Figure S1. Total ion chromatogram by positive mode electrospray ionization mass (A). ^{1}H-NMR spectrum with expansion of AC extract in DMSO-*d6* (B).

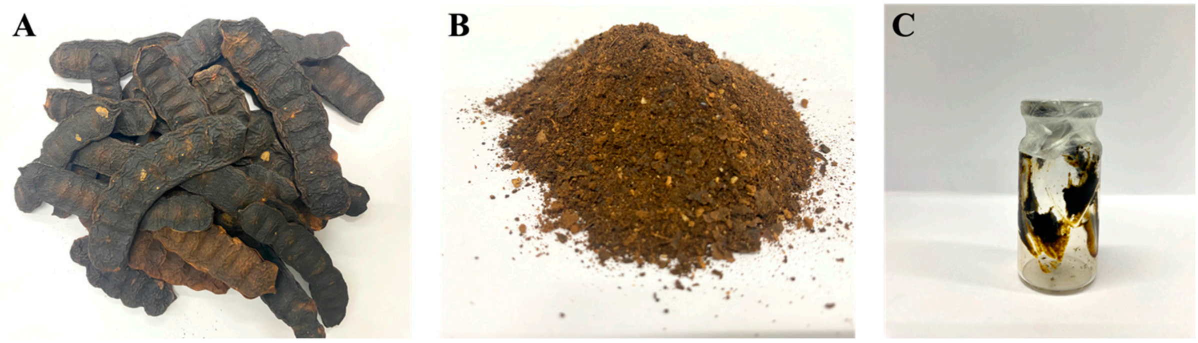

Figure S2. The dried pods of *Acacia concinna* (A) were ground into powder (B) for extraction. The crude extract of *A. concinna* (C) was kept in the refrigerator, in the dark, until used in the experiment.

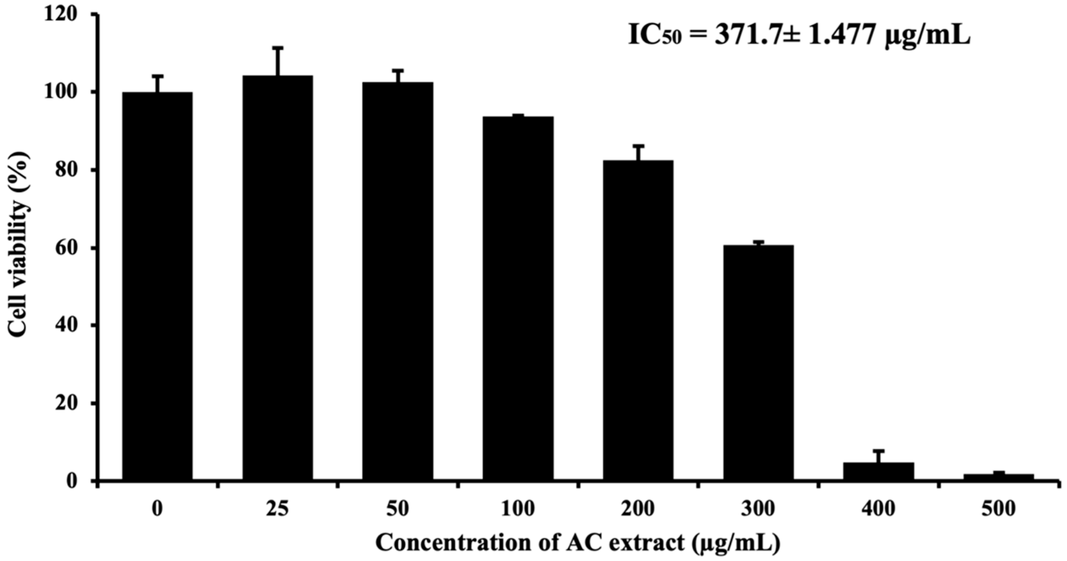

Figure S3. Effect of AC extract on cell viability in HaCat cells by MTT assay. The cell line was treated with various concentrations of AC extract for 24 h. The results were the mean values ± SD.
